# Hyperactivity and attention deficit syndrome in obstructive sleep apnea syndrome: is there improvement with surgical management?

**DOI:** 10.1016/S1808-8694(15)30045-8

**Published:** 2015-10-19

**Authors:** Silke Anna Thereza Weber, Arlindo Cardoso Lima Neto, Fernando José de Souza Ternes, Jair Cortez Montovani

**Affiliations:** a MSc, Assistant Professor.; b 1^st^ year otolaryngology resident.; c 2^nd^ year otolaryngology resident.; d Associate Professor, Assistant PhD Professor.

**Keywords:** impulsivity, hyperactivity, adenotonsillectomy

## Abstract

Neuropsychological disorders are frequently associated with obstructive ventilatory disorders (OVD).

**Aim:**

To analyze the incidence of neuropsychological disorders in Brazilian children with OVD, using a screening questionnaire and to compare the answers given before and after surgery.

**Patients and Methods:**

We studied 30 children with clinical diagnosis of OVD. The children were divided into 3 groups: group I, children aged 4 to 7; group II, from 8 to 10; and group III, children over 11. The applied questionnaires were answered by the parents or tutors, and comprised 30 questions, 10 for each disorder: attention deficit, hyperactivity and impulsivity. The children were diagnosed with one of the disorders when presented 3 or more positive answers. The follow up interview occurred 6 months after adenotonsillectomy.

**Results:**

There was a predominance of male gender (60.6%) over female gender (39.4%). Group II presented the highest number of significant changes, with reductions raging from 87.5% to 33.3% of patients with attention deficit, 75% to 50% of the hyperactive patients, and 50% to 33% of the impulsive patients.

**Conclusion:**

There was neuropsychological improvement after the surgery, which occurred mainly in the children from group II. More interaction among health professionals is necessary when diagnosing and following up similar cases.

## INTRODUCTION

Obstructive ventilatory disorders (OVD) are air flow alterations due to total or partial collapse of the upper airways. It may manifest as snoring, oral breathing, apneas or hypopneas. Its most severe form, the obstructive sleep apnea syndrome (OSAS) is a disorder characterized by repeated episodes of upper airway obstruction associated to hemoglobin desaturation^1^. Full night polysomnography is the gold standard exam for OSAS diagnosis. Its prevalence in children vary from 0.7 to 3% in different epidemiological studies^1^, ^2^, ^3^. The incidence peak is observed in pre-school age children, age range in which upper airway obstructions are more common because of upper airway obstruction due to hypertrophy of pharyngeal tonsils and adenoid^4^. Besides oral breathing, other OSAS symptoms are: snoring, respiratory pauses, breathing impairment, restless sleep, sweating, nocturnal enuresis^5^, and neuropsychomotor disorders.

Many papers report that, the main cognitive deficit in OSAS children are related to concentration and attention, essential functions for schooling^6^, ^7^, ^8^.

It is increasingly more common to see children with diagnosis of attention deficit and hyperactivity disorder (ADHD), but we rarely imagine that such behavior disorder may be related to primary snoring or obstructive sleep apnea. Notwithstanding, there is enough evidence of hyperactivity and sleep respiratory disorders coexisting in 20% to 30% of the cases^9^, ^10^, ^11^.

## LITERATURE REVIEW

Gozal^6^ assessed the prevalence of sleep respiratory disorders in 782 children with poor schooling performance in the first grade of basic teaching in public American schools. The author interviewed the parents about respiratory signs and symptoms of their children and, later on, the children underwent pulse oximetry study and capnogram during the night. A ≥5 score in the symptoms questionnaire without oxihemoglobin desaturation finding, nor hypercapnia was the diagnostic criteria for primary snoring. In cases of scores ≥5 in the symptoms questionnaire, two or more desaturation episode per hour of sleep (SaO2<95%) and/or hypercapnia (nocturnal PaCO2 8mmHg above daily PaCO2 for over 60% of the sleeping time) were diagnosed as sleep respiratory disorders. In this sample, the prevalence of primary snoring was of 22.2%. Twenty four children with sleep respiratory disorders were then referred to adenotonsillectomy, and their school grades had a significant improvement in the year following surgery. The paper showed a negative impact of sleep respiratory disorders in learning^6^. Blunden et al.^7^ assessed the cognition of 16 children from 5 to 10 years of age who were referred for snoring treatment and 16 normal controls of the same age. They observed that the snoring children had attention deficit and concentration problems even in the absence of daily sleepness^7^. Gozal and Pope Jr.^8^ retrospectively assessed the possible association between school performance of teenagers and a past history of frequent nocturnal snoring when they were between 2 and 6 years of age. 1588 adolescents between 13 and 14 years enrolled in public schools of an American county were selected. Of these, 797 were among the 25% of students with the worst grades, and 791 among the 25% with the best grades. The incidence of nocturnal snoring was of 12.9% in the group of bad students and only 5.1% in the group of good students. Although we may not ascertain whether or not these teenagers had primary snoring or OSAS during childhood, we can see that the repercussions of the sleep respiratory disorders on schooling performance may last for a long time8. O’Brien et al.^11^ evaluated the relationship between sleep respiratory disorders, attention deficit and hyperactivity (ADHD) in 5728 children between five and seven years of age enrolled in American public schools, of whom 418 were hyperactive. Among the total number of students, 11% presented high and frequent nocturnal snoring. Among the hyperactive the rate of snorers went up to 23%. Besides snoring, symptoms of bruxism (clenching the teeth) and restless sleep were also more frequently found in hyperactive children. The authors submitted 71 snorer children with severe ADHD and 39 normal controls to a full night polysomnography in a sleep lab. In the former group, 20% of the children had obstructive sleep apnea (IAH≥5), greater latency and less REM sleep duration. The physiopathology of this relationship: obstructive sleep apnea-hyperactivity is still uncertain. It is possible that chronic OSAS-induced hypoxemia causes biochemical alterations in the pre-frontal lobe, thus affecting the dopaminergic, adrenergic and glutamate pathways^11^. There were many attempts to withdraw the patients with neuropsychological disorders through protocols and questionnaires. Since 1994, we have in effect a scale published in the Statistical Manual of Mental Disorders - 4^th^ Edition (DMS-IV), divided in 30 questions about the behavior of minors, there are four answering alternatives: “never”, “a little”, “very much” or “too much”. Questions of which answers are “very much” or “too much” are considered “positives”. The thirty questions are divided in 3 groups of 10, and the first is made up of questions pertaining to the attention/concentration deficit, the second is related to hyperactivity and the third to impulsiveness, which are the three possible ADHD presentations according to the manual.

## OBJETIVE

The goal of this study was to analyze the prevalence of neuropsychological disorders (hyperactivity disorder, attention and concentration deficit disorder and impulsiveness disorder) in children with Obstructive Ventilatory Disorder (OVD) through a specific questionnaire, comparing the pattern of answers before and after adenotonsillectomy.

## MATERIALS AND METHODS

30 children between 4 and 13 years of age with clinical diagnosis of OVD, under follow up at the Department of Sleep Disorders of the Otolaryngology Department - HC-UNESP/Botucatu. The clinical diagnosis of Obstructive Ventilatory Disorder was considered when the patient presented frequent nocturnal snoring, constant oral breathing and restless sleep. We may also have respiratory pauses seen, parasomnia, nocturnal excessive sweating, enuresis and awakenings. Some children were unable to complete the full night polysomnography, and that is why we did not use the diagnosis of OSAS but that of OVD.

Otolaryngology exam, including nasofibroscopy, presented obstructive hypertrophy of pharyngeal tonsils and adenoids, and surgery was indicated. Of the 30 children involved in the study, 22 returned for the second visit and interview six months after surgery (adenotonsillectomy). All participants went through a hearing evaluation through clinical, otological and audiometric and immitanciometry exams, those with any alteration were excluded. We also excluded those children with neuropsychomotor development delays, those with neurological diseases, mental retardation and genetic syndromes.

The indication of adenotonsillectomy was made based on patient complain, clinical exam and nasofibroscopy (Storz, 3.4cm diameter bronchoscope) compatible with obstruction of at least 80% of the choana lumen by adenoids, besides oropharynx obstruction caused by the tonsils after Müler's maneuver. The children were divided in 3 groups according to their age range. Group I included the children from 4 to 7 years, considered of pre-school age; group II from 8 to 10 years – starting school activities; and group III with children above 11 years of age, when there are more complex school demands. The patient's guardian answered a screening questionnaire of hyperactivity disorders, attention deficit and impulsiveness, adapted from the DSM – IV protocol^10^, ^11^, in such a way that the same person answered it before and after the surgical procedure.

**Graph 1:**
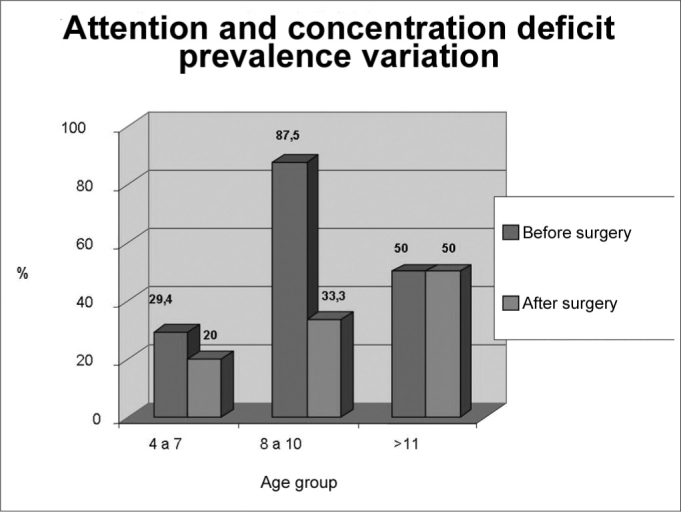
Attention and concentration deficit disorder prevalence according to age groups, before and after adenotonsillectomy (n=22)

**Source:** Authors’ data base

**Graph 2:**
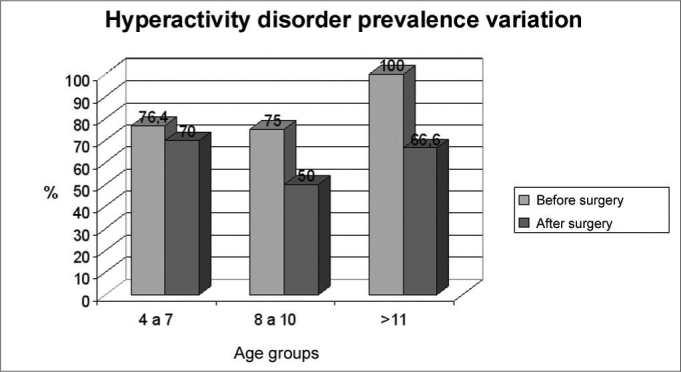
Hyperactivity disorder prevalence according to age groups before and after adenotonsillectomy (n=22)

**Source:** Authors’ data base

**Graph 3:**
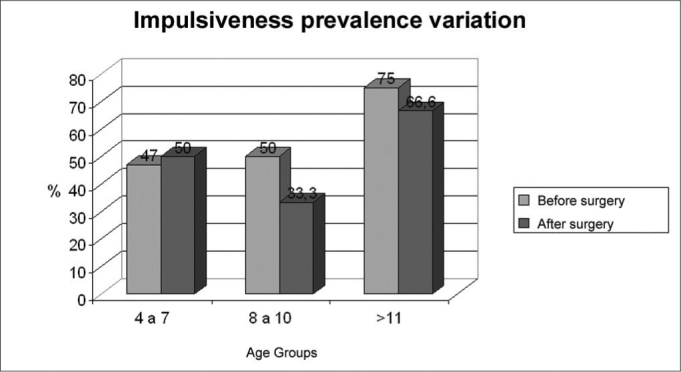
Impulsiveness disorder prevalence according to age groups, before and after adenotonsillectomy. (n=22)

**Source:** Authors’ data base

**Chart 1:**
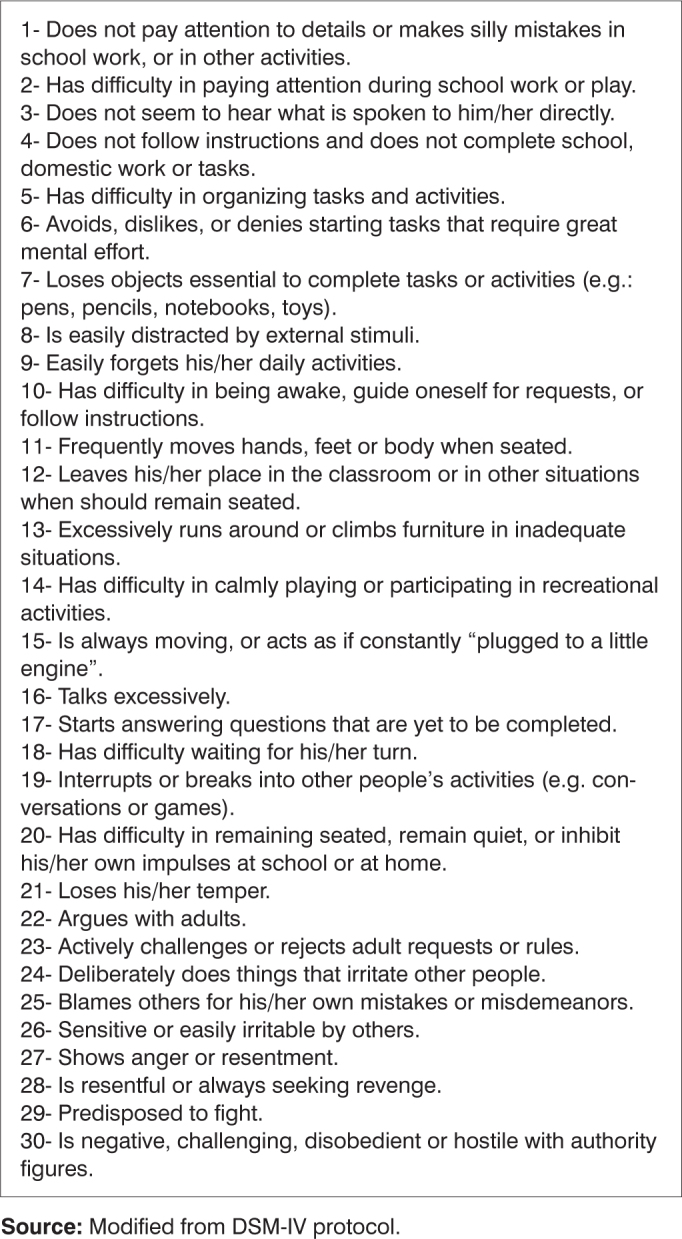
Attention/Concentration deficit disorders related questions, hyperactivity and impulsiveness.

**Source:** Modified from DSM-IV protocol.

The protocol has 3 blocks of 10 questions related to signs of attention and concentration deficits, hyperactivity and impulsiveness, with quantitative answer grading of 1-absent (“never”); 2- not so frequent (“little”); 3- moderately frequent (“very much”); 4- very frequent (“too much”) (Chart 1). When three or more questions received a positive answer of moderate and/or very frequent, the screening test was considered suggestive for neuropsychological disorder. The children with positive screening test, even after surgery, were referred to a specialized neuropediatrics department in this same institution.

For statistical assessment of the results, we used the McNemar method based on the construction of 2x2 tables with the age groups and the disorder to be studied. This method allows the comparison of two moments in the same population, without the need to have a control group.

## RESULTS

We studied 30 children with ages varying from 4 to 13 years, 8 years and 4 months in average. According to the age-related division, 15 children (50%) in group I, 8 children (26.6%) in group II and 7 children (23.3%) in group III. As to the gender distribution, there was a prevalence of boys (60.6%) in relation to girls (394%). This male dominance was observed in the ages between 4 and 7 years (76.4 %), with an inversion between 8 and 10 years (37.5% boys: 62.5% girls), and in those older than 11 years (50% boys: 50% girls).

In group I, three or more positive answers were found in 76.4% of the questions regarding hyperactivity, 47% regarding impulsiveness and 29.4% regarding attention and concentration deficit. After surgery the values were: 70% (P=1.0), 50% (P=1.0) and 20% (P=0.48) respectively.

In group II we found 87.5% of children with attention deficit, and this reduced to 33.3% (P=1.0) after adenotonsillectomy. The rate of hyperactive disorder of 75% dropped to 50% (P=0.48), and the 50% of impulsiveness dropped down to 33.3% (P=1.0).

In group III, the frequence of neuropsychological disorders before and after surgery was of 100% to 66.6% (P=1) of hyperactive, 75% to 66.6% (P=1) of impulsive and 50% and 50% (P=0.24) of children with attention and concentration deficit.

This data is shown on Charts 1, 2 and 3.

The most frequently positive questionnaire items in the three age groups, before and after surgery, are depicted on Table 1.

**Table 1:** Number of more frequently positive issues in the questionnaire among the different age ranges, before and after adenotonsillectomy.

	Concentration and attention deficit		Hyperactivity		Impulsiveness	
	Before	After	Before	After	Before	After
Group I (4 - 7 years)	3 e 7 (41%)	8 (30%)	12 e 16(58,8%)	16 (70%)	26 (58,8%)	21 (70%)
Group II (8 - 10 years)	1 e 7 (75%)	1 (50%)	11 (75%)	15 e 16 (50%)	26 (75%)	21, 24 e 26 (50%)
Group III (>11 years)	3 (75%)	8, 9 e 10 (50%)	17 (62,5%)	16 e 19(50%)	21 e 26 (87,5%)	21, 22, 23 e 26 (66,6%)

## DISCUSSION

In this study we found a dominance of children in the age range of 4 to 7 years of age, when we have the highest incidence of palatine tonsils and adenoids, obstruction the upper air ways^4^. Of these, 76.4% were male. A male dominance in OVD patients is frequently found in the literature^9^, ^12^, and it could be related to a higher incidence of upper airway infections and craniofacial characteristics. After the growth spurt at 7 years of age, we no longer find this prevalence, because the OVD causes are more related to nasal and alergic factors amongst others, and no longer to tonsillar and adenoid hypertrophy. Similar to what the literature is showing, we noticed an improvement in attention disorders and hyperactivity with surgical treatment. We observed this happening in this study, specially in school age children (8 to 11 years) even in a six month time gap, strongly suggesting that the improvement in these children sleep patterns have contributed to better school performance. In the pre-school years group, the reduction in neuropsychological disorders may have been less significant due to a bias present in these children's social environment: this age range has less demanding schooling and from parents’ stand point, and this potentially reduces the benefits that the adopted treatment can offer these children.

In their turn, the children above 11 years improved, probably because 6 months would be little time for such change, specially when dealing with older children, who suffered OVD for longer, already suffering sequels by this chronic functional alteration.

It is true that such findings are only numerically suggestive, because P varied between 0.24 and 1.0, probably due to the not so large sample size. McNemar statistical method was used because it analyses the impact of a given phenomenon in the same population, comparing this population before and after the event under study. On the other hand, many patients presented significant improvement in their response pattern, but not enough to take them off the group of probable carriers of the disorder. In other words, if an interviewee is positive in 8 of the 10 questions about a particular disorder, but in the second time he/she answers the questionnaire is positive in only 3, there is no subtraction in the disorder, because it is still considered positive in that issue. Of course, if such clinical improvements were at the reach of the statistical method, there would be greater significance. Observing the most frequent issues before and after adenotonsillectomy (Table 1), we notice that some of them repeat themselves in all ages, such as the ones in numbers 3, 7, 8, 16, 21 and 26. It is interesting to highlight that all these items have very direct and easily understandable headlines, certainly more adequate to be given to less educated populations.

## CONCLUSIONS

We concluded that the frequence of neuropsychological disorders in OVD children is high, easily seen in younger children through hyperactivity and, in older children, through the other two types: attention/concentration deficit and impulsiveness, affecting their schooling performance and their socialization.

This phenomenon suggests that there is improvement in neuropsychological disorders in OVD children who undergo adenotonsillectomy.

The parents/guardians, teachers and health care professionals should pay more attention towards neuropsychological disorders with possible relationship with snoring and apneas, as well as greater integration among medical specialties for this end.
